# Mechanisms Mediating the Combined Toxicity of Paraquat and Maneb in SH-SY5Y Neuroblastoma Cells

**DOI:** 10.1021/acs.chemrestox.3c00389

**Published:** 2024-07-26

**Authors:** Suzana da Silva, Carolina de Lima da Costa, Aline Aita Naime, Danúbia Bonfanti Santos, Marcelo Farina, Dirleise Colle

**Affiliations:** † Department of Clinical Analyses, Federal University of Santa Catarina, Florianopolis 88040-900 Santa Catarina, Brazil; ‡ Department of Biochemistry, Federal University of Santa Catarina, Florianopolis 88040-900 Santa Catarina, Brazil

## Abstract

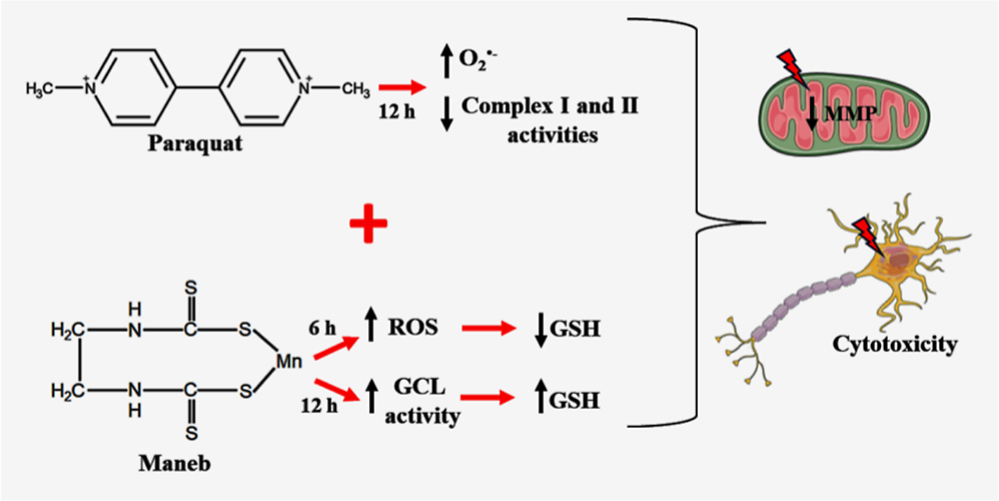

Epidemiological and experimental studies have demonstrated that combined exposure to the pesticides paraquat (PQ) and maneb (MB) increases the risk of developing Parkinson’s disease. However, the mechanisms mediating the toxicity induced by combined exposure to these pesticides are not well understood. The aim of this study was to investigate the mechanism(s) of neurotoxicity induced by exposure to the pesticides PQ and MB isolated or in association (PQ + MB) in SH-SY5Y neuroblastoma cells. PQ + MB exposure for 24 and 48 h decreased cell viability and disrupted cell membrane integrity. In addition, PQ + MB exposure for 12 h decreased the mitochondrial membrane potential. PQ alone increased reactive oxygen species (ROS) and superoxide anion generation and decreased the activity of mitochondrial complexes I and II at 12 h of exposure. MB alone increased ROS generation and depleted intracellular glutathione (GSH) within 6 h of exposure. In contrast, MB exposure for 12 h increased the GSH levels, the glutamate cysteine ligase (GCL, the rate-limiting enzyme in the GSH synthesis pathway) activity, and increased nuclear Nrf2 staining. Pretreatment with buthionine sulfoximine (BSO, a GCL inhibitor) abolished the MB-mediated GSH increase, indicating that MB increases GSH synthesis by upregulating GCL, probably by the activation of the Nrf2/ARE pathway. BSO pretreatment, which did not modify cell viability per se, rendered cells more sensitive to MB-induced toxicity. In contrast, treatment with the antioxidant *N*-acetylcysteine protected cells from MB-induced toxicity. These findings show that the combined exposure of SH-SY5Y cells to PQ and MB induced a cytotoxic effect higher than that observed when cells were subjected to individual exposures. Such a higher effect seems to be related to additive toxic events resulting from PQ and MB exposures. Thus, our study contributes to a better understanding of the toxicity of PQ and MB in combined exposures.

## 1 Introduction

Parkinson’s disease (PD) is a neurodegenerative condition characterized by the degeneration of dopaminergic neurons in the substantia nigra pars compacta (SNpc) and accumulation of protein clumps named Lewy bodies (LBs).^1^ Dopaminergic neuronal degeneration in the SNpc leads to a reduction of dopamine in the striatum, resulting in motor symptoms including slow movement (bradykinesia), muscle stiffness (rigidity), and tremors at rest.^2^ Although genetic factors contribute to the development of PD, most of the cases are sporadic (∼90%) with an undetermined etiology. While the precise cause of PD is still unclear, aging is the most significant risk factor for the disease. However, epidemiological studies have indicated that occupational exposure to environmental toxicants such as metals, solvents, and pesticides might be linked to PD physiopathogenesis.^3^ Over the past two decades, considerable attention has been given to the impact of pesticide exposure in rural areas with Parkinsonism,^4−6^ further supporting the hypothesis that these environmental chemicals contribute to the loss of dopaminergic neurons in the SNpc, stimulating PD development/progression.

Several lines of evidence have linked the risk of developing PD to the exposure to some pesticides like paraquat (PQ) and maneb (MB).^4−6^ PQ is a nonselective herbicide that remains extensively used in Third World countries; its toxicity is linked to the ability to induce redox cycling, which produces superoxide anion radicals (O_2_^•–^) and leads to oxidative stress.^7^ The administration of PQ systemically in mice results in degeneration of dopaminergic neurons in the SNpc.^8,9^ The fungicide MB has also been identified as a potential risk factor for developing PD. MB exposure reduces locomotor activity^10^ and induces selective dopaminergic neurodegeneration^11^ in rodent models. The mechanisms mediating MB-induced toxicity seem to be related to mitochondrial dysfunction,^12^ alterations in metabolic pathways,^13^ neurotransmitter system disturbance,^14^ and disruption of redox circuits.^15^ Of note, it has been reported that MB potentiate PQ toxicity in *in vivo*(16) and *in vitro* experimental conditions.^17^ Exposure to PQ plus MB in combination causes disruption of mitochondrial membrane potential (MMP) and induced oxidative stress in dopaminergic-like neurons.^18^ Moreover, exposure to PQ plus MB induces ferroptosis in SH-SY5Y cells, which was linked to NADPH oxidase activation.^19^

In fact, simultaneous exposures to PQ and MB (PQ + MB) induce more pronounced dopaminergic neurotoxicity than that observed when the pesticides are administered isolated, resembling a PD phenotype in rodent models.^16,20,21^ These findings have attracted significant attention because PQ and MB are frequently used together in the same geographic regions, and epidemiologic evidence suggests that populations exposed to both pesticides may face an elevated risk of developing PD.^4^

Humans are likely to be exposed to multiple pesticides (either simultaneously or sequentially) in their residential and occupational environments, suggesting that exposure to mixtures of these agents may exert additive or synergistic effects.^4,22−24^ Even though the toxicity induced by exposures to individual pesticides has been widely studied, combined exposures to different pesticides are less frequently studied, particularly under realistic scenarios. However, evidence has shown that combined exposures to pesticides have negative impacts on health and could potentially elevate the risk of developing diseases, including neurodegenerative disorders.^22,23,25^

As previously mentioned, experimental animal and *in vitro* studies have shown that the simultaneous exposure to PQ and MB results in more pronounced harmful effects compared to exposures to each pesticide individually.^16,17,20^ Although some lines of evidence have pointed to potential additive toxic effects of PQ + MB,^17^ the mechanism(s) of their combined toxicity remains unknown. Therefore, we investigated the neurotoxicity induced by the individual and/or combined exposure to the pesticides PQ and MB in undifferentiated SH-SY5Y neuroblastoma cells in order to provide mechanism(s) mediating neurotoxicity induced by the combination of pesticides.

## 2 Material and Methods

### 2.1 Chemicals

PQ, MB, β-nicotinamide adenine dinucleotide reduced dipotassium salt (NADH), buthionine sulfoximine (BSO), *N*-acetylcysteine (NAC), reduced glutathione (GSH), 3-(4,5-dimethylthiazol-2-yl)-2,5-diphenyltetrazolium bromide (MTT), 2,7-dichlorofluorescein diacetate (DCFH-DA), 5,5′-dithiobis(2-nitrobenzoic-acid) (DTNB), 5,5′,6,6′-tetrachloro-1,1′,3,3′-tetraethyl-imidacarbocyanine iodide (JC-1), lactic desidrogenase, pyruvate quinase, and dimethyl sulfoxide (DMSO) were purchased from Sigma-Aldrich (St. Louis, MO, USA). Fetal bovine serum (FBS) and Dulbecco’s modified Eagle’s medium F12 (DMEM F12) were purchased from Gibco Life Technologies (Carlsbad, CA). Rabbit polyclonal anti-Nrf2 antibody (SAB4501984) was purchased from Sigma-Aldrich. Alexa fluor 488 goat antirabbit IgG secondary antibody (A-11008, Invitrogen) was purchased from Thermo-Fisher Scientific.

### 2.2 Cell Culture

SH-SY5Y human neuroblastoma cells (ATCC CRL-2266), obtained from the Rio de Janeiro Cell Bank (RJ, Brazil), were cultured as monolayers in polystyrene dishes. Cells were maintained in DMEM-F12 supplemented with 2 mM glutamine, 100 units/mL penicillin, 100 μg/mL streptomycin, and 10% of FBS at 37 °C in a humidified atmosphere containing 5% CO_2_. Cell splitting was performed every 3–4 days at 70–80% confluency and used between the fifth and 15th passages. The culture medium was refreshed every 3 days. Depending on the experimental procedure, cell suspensions were seeded in either 100 × 20 mm Petri dishes or multiwell plates (96, 24, 12, or 6 wells). After 48 h in culture, cells were exposed to the pesticides or other treatments, as described below.

### 2.3 Cell Treatments

PQ was dissolved in phosphate buffer saline (PBS) and MB was prepared in DMSO, whose final concentration in cells did not exceed 0.01%. NAC and BSO were dissolved in ultrapure water. Initially, SH-SY5Y cells were treated with vehicle (PBS or DMSO), PQ (10, 30, 100, 300, 1000, and 3000 μM), or with MB (1, 3, 10, 15, 20, and 30 μM) for 24 h. In different experimental protocols, cells were exposed to 100 μM PQ and 10 μM MB alone or in combination (PQ + MB) for 6, 12, 24, 48, or 72 h. In parallel experiments, cells were pretreated with 25 μM BSO or 500 μM NAC and then exposed to the pesticides.

### 2.4 Cell Viability Assays

Cells were plated into 96 well-plates at equal density (2.0 × 10^4^ cells/well). 48 h after seeding, SH-SY5Y cells were incubated with vehicle (PBS or DMSO), PQ (10–3000 μM), or MB (1–30 μM) for 24 h or treated with 100 μM PQ and 10 μM MB alone or in combination (PQ + MB) for 6, 12, 24, 48, or 72 h. In parallel, cells were incubated with BSO (25 μM) for 24 h or NAC (500 μM) for 1 h and then exposed to 100 μM PQ and/or 10 μM MB for 48 h.

After treatments, cell viability was assessed using two distinct assays that evaluate different aspects of cellular function. The MTT assay, originally described by Mosmann,^26^ measures the metabolic activity of viable cells by assessing their ability to convert MTT (3-(4,5-dimethylthiazol-2-yl)-2,5-diphenyl-tetrazolium bromide) into a purple formazan product through mitochondrial dehydrogenases. After the treatments, the medium was aspired, and cells were incubated with 0.5 mg/mL MTT for 1.5 h at 37 °C. The formazan product was solubilized by DMSO, absorbance was quantified using spectrophotometry (Infinite M200 Microplate Reader, Mannedorf, Switzerland) at 540 nm, and results were expressed as percentage of control values (cells treated with vehicle).

The lactate dehydrogenase (LDH) release assay was conducted to assess plasma membrane integrity following a previously established protocol.^27^ After exposure to pesticides, 10 μL of 2% triton X-100 (0.2% final concentration) was added to cells designated as the positive control (presenting 100% of cell death). After 15 min of incubation at 37 °C, 50 μL of culture medium was removed and transferred to a new 96-well plate. To this plate, containing only the culture medium, 200 μL of reaction mix (0.5 M sodium phosphate buffer pH 7.4 containing 4.7 mM sodium bicarbonate, 2.08 mM sodium pyruvate, and 0.36 mM NADH) was added. The absorbance was determined in a microplate reader (Infinite M200 Microplate Reader, Mannedorf, Switzerland) at 340 nm every 15 s for 150 min. Results were expressed as a percent of LDH released, where cells treated with 2% Triton X-100 were considered as 100% (100% of cell death). All experiments were performed in triplicate.

### 2.5 Measurement of Reactive Oxygen Species Production

Intracellular reactive oxygen species (ROS) production was detected using the nonfluorescent cell-permeable compound 2′,7′-dichlorodihydrofluorescein diacetate (DCFH-DA).^28^ Inside the cells, DCFH-DA is hydrolyzed by esterases to form the membrane impermeable product DCFH, which is trapped inside the cells. DCFH reacts with intracellular ROS to produce the fluorescent compound 2′,7′-dichlorofluorescein (DCF). Cells were plated into 12 well-plates at equal density (1.75 × 10^5^ cells/well). SH-SY5Y cells were incubated with vehicle (PBS or DMSO) or with 100 μM PQ and 10 μM MB alone or in combination (PQ + MB) for 6 or 12 h. At the end of exposure, cells were washed with a Hanks’ balanced salt solution (HBSS) and then incubated with 3 μM DCFH-DA for 30 min at 37 °C in HBSS. After the incubation, cells were washed with PBS and then incubated with trypsin solution 1× in PBS for 3 min at 37 °C. Next, cells were harvested from the plate and collected to Eppendorfs containing 1% FBS solution in HBSS and then centrifuged at 1200 rpm for 3 min at room temperature. After centrifugation, the supernatants were discarded, and the pellets were washed twice with HBSS. The fluorescence intensity was measured with a BD FACS Canto II flow cytometer (BD Biosciences, CA, USA). Results were expressed as the percentage of control (cells treated with vehicle) fluorescence intensity.

### 2.6 Measurement of Superoxide Anion (O_2_^•–^) Production

The production of superoxide anions in cells exposed to pesticides was assessed by using dihydroethidium (DHE). This assay relies on the oxidation of DHE by O_2_^•–^ to form the fluorescent compound ethidium.^29^ Cells were plated into 24-well plates at equal density (1.25 × 10^5^ cells/well) and exposed to vehicle (PBS or DMSO) or to 100 μM PQ and 10 μM MB alone or in combination (PQ + MB) for 6 or 12 h. After pesticide exposure, the media was removed, and cells were washed once with HBSS and then incubated with DHE (10 μM) in HBSS for 30 min at 37 °C. Then, cells were washed with HBSS, and cellular fluorescence was recorded with excitation at 488 nm and emission at 585 nm in a fluorimetric microplate reader (Infinite M200 Microplate Reader, Mannedorf, Switzerland). Results were expressed as the percentage of control (cells treated with vehicle) fluorescence intensity.

### 2.7 Measurement of Mitochondrial Membrane Potential (MMP)

MMP was determined using the lipophilic cationic probe fluorochrome JC-1.^30^ Under normal MMP conditions, JC-1 forms aggregates that produce red to orange fluorescence with an emission peak at 588 nm. When the membrane potential is lost, the dye shifts to its monomeric form, resulting in green fluorescence with an emission peak at 530 nm. SH-SY5Y cells were seeded into 24 well-plates (1.25 × 10^5^ cells/well) and exposed to vehicle (PBS or DMSO) or to 100 μM PQ and 10 μM MB alone or in association (PQ + MB) for 6 or 12 h. The compound FCCP (carbonyl cyanide 4-(trifluoromethoxy)phenylhydrazone (10 μM) for 3 h was used as a positive control, which decreased the MMP to 30% of the control values. After pesticide exposure, the media was removed, and cells were washed once with HBSS and then incubated with JC-1 (5 μM) for 20 min at 37 °C. Then, cells were washed with HBSS, and JC-1 fluorescence intensity was measured for J-aggregates (red, excitation at 550 nm and emission at 600 nm) and monomers (green, excitation at 485 and emission at 535), respectively, using a fluorimetric microplate reader (Infinite M200 Microplate Reader, Mannedorf, Switzerland). The MMP was determined from the ratio of fluorescence intensity to red and green fluorescence. Results were expressed as the percentage of the control (cells treated with vehicle).

### 2.8 Measurement of the Mitochondrial Respiratory Chain Complexes Activities

Cells were seeded at a density of 2.3 × 10^6^ cells on 100 mm dishes. SH-SY5Y cells were treated with vehicle (PBS or DMSO) or with 100 μM PQ and 10 μM MB alone or in combination (PQ + MB) for 12 h. Following the treatments, cells were rinsed and harvested in 500 μL of 4.4 mM potassium phosphate buffer pH 7.4, containing 0.3 M sucrose, 5 mM MOPS, 1 mM EGTA, and 0.1% bovine serum albumin, and then centrifuged at 1000 × *g* for 10 min at 4 °C. The supernatants were collected and stored at −70 °C for enzyme activity assays. NADH dehydrogenase (complex I) activity was assessed by measuring the rate of NADH-dependent ferricyanide (FeCN) reduction, as previously described.^31,32^ For complex I activity, 50 μL of supernatants were mixed with 40 μM rotenone and 0.5 mM FeCN in a potassium phosphate buffer (100 mM, pH 7.4), followed by the addition of 0.2 mM NADH. Complex I activity was analyzed in a Microplate Reader (Infinite M200 Microplate Reader, Mannedorf, Switzerland) at 420 nm for 5 min and calculated as nmol/min/mg protein.

The activity of succinate-2,6-dichloroindophenol (DCIP)-oxidoreductase (complex II) was assessed according to a previously standardized protocol.^33^ Samples (30 μL) were incubated with a potassium phosphate buffer (62.5 mM, pH 7.4) in the presence of 8 mM sodium succinate and 5 μM 2,6-DCIP at 37 °C for 20 min. After the incubation, 40 μM rotenone, 2.5 mM sodium azide, and 25 μM 2,6-DCIP were added in the incubation medium. The activity of complex II was determined by following the decrease in absorbance due to the reduction of 2, 6-DCIP at 600 nm for 5 min (Infinite M200 Microplate Reader Mannedorf, Switzerland) and calculated as nmol of 2,6-DCIP reduced. min^–1^ mg protein^–1^. Results were expressed as the percentage of control (cells treated with vehicle).

### 2.9 Assessment of GSH Content

GSH content was assessed as nonproteic thiols (NPSH), as previously described.^34^ Approximately 90% of the total thiols from nonprotein sources represent GSH.^35^ In addition, GSH was also assessed by the method of Tietze 1969, which accurately determines the total GSH content in samples without significant interference from other thiol compounds.^36^ SH-SY5Y cells were seeded into 6 well-plates at equal density (4.0 × 10^5^ cells/well). Cells were exposed to the vehicle (PBS or DMSO) or to 100 μM PQ and 10 μM MB alone or in combination (PQ + MB) for 6 or 12 h. In another experiment, cells were incubated with 25 μM BSO for 10 h and then were exposed to 10 μM MB for 12 h.

For determination of GSH as NPSH, cells were washed and collected in 150 μL of PBS buffer (pH 7.4) containing 0.05% Triton X-100, then mixed with a 10% trichloroacetic acid solution. After centrifugation (5000×*g* at 4 °C for 10 min), the protein pellet was discarded. Free thiol groups in the clear supernatant were determined by reacting with 10 mM DTNB. Absorbance was measured in 412 nm (Infinite M200 Microplate Reader, Mannedorf, Switzerland), and GSH was used as a standard. Results were expressed as the percentage of control (cells treated with vehicle).

For determination of GSH content by the method of Tietze, cells from 6-well plates were rinsed and collected in 200 μL of PBS buffer (pH 7.4). The cells were then mixed in a cold solution containing 10 mM HCl and 10% 5-sulfosalicylic acid dihydrate (SSA). Samples were centrifuged at 12,000*g* at 4 °C for 5 min, and the clear supernatants were diluted 1/3 with 3.3% SSA. Diluted samples were mixed in KPE buffer (0.1 M potassium phosphate +5 mm EDTA pH 7.4), containing 0.22 mM DTNB, 0.3 mM NADPH, and 0,12 μg of GSH reductase. Absorbance was monitored at 412 nm during 4 min at 30 °C. The GSH content in each sample was determined using a GSH standard curve (0–25 nmol) and normalized to the protein content. The results, expressed as nmol of GSH/mg protein, were then presented as a percentage relative to the control group (cells treated with vehicle).

### 2.10 Assessment of Glutamate Cysteine Ligase Activity

The enzymatic activity of glutamate cysteine ligase (GCL) was measured following the protocols established by Seelig and co-workers^37^ with slight modifications.^38^ SH-SY5Y cells were seeded into 6 well-plates at equal density (4.0 × 10^5^ cells/well). Cells were exposed to vehicle (DMSO) or 10 μM MB for 6 or 12 h. In a parallel experiment, cells were incubated with 25 μM BSO for 10 h and then were exposed to 10 μM MB for 12 h. After the incubation protocol, cultures were washed and collected in 300 μL of lyses buffer (1 M Tris/HCl, 50 mM MgCl_2_, 0.05% Triton X-100, pH 8.0) and centrifuged at 12,500 rpm for 30 min at 4 °C. Samples were mixed in a Tris–HCl buffer (0.1 M Tris–HCl, 1.15 nM KCl, 0.15 M MgCl_2_, 0.15 M EDTA) pH 8, containing 0.5 mM l-glutamate, 0.5 mM l-α-aminobutyrate, 0.25 mM Na_2_-ATP, 0.2 mM NADH, and 17 μg of pyruvate kinase/LDH. GCL activity was assessed by monitoring the NADH oxidation at 340 nm during 10 min in a microplate reader (Infinite M200 Microplate Reader, Mannedorf, Switzerland). The results were presented as a percentage relative to the control group (cells treated with the vehicle).

### 2.11 Immunofluorescence Assay

The effect of MB on Nrf2 nuclear translocation was evaluated by immunofluorescence. Cells were seeded in coverslips precoated with poly-d-lysine in 12 well-plates at a density of 1.75 × 10^5^ cells/well. Cells were exposed to 10 μM MB or vehicle for 6 h. *tert*-Butylhydroquinone (TBQ; 10 μM for 6 h) was used as a positive control. Cells were fixed with 4% paraformaldehyde for 15 min and permeabilized with 0.3% triton X-100 for 10 min at room temperature. Next, they were blocked with 5% goat serum in PBS buffer for 1 h and washed with 0.1% (v/v) Tween 20-PBS (PBS-T20) for 5 min. Cells were incubated overnight at 4 °C with a rabbit polyclonal anti-Nrf2 antibody (1:500) in a blocking solution. Following three 5 min washes with PBS-T20, the cells were incubated for 1 h at room temperature with an Alexa Fluor 488 goat antirabbit IgG secondary antibody (1:400). Cells were then incubated for 10 min with Hoechst 33258 for nuclei staining. The images were captured from eight randomly selected fields by using a fluorescent microscope (Olympus BX41).

### 2.12 Protein Determination

The total protein content in samples used for the determination of GSH levels, GCL, and complex I and II activities was quantified by the Lowry method^39^ using the Folin and Ciocalteu’s phenol reagent (Sigma-Aldrich). The protein content in each sample was calculated based on a bovine serum albumin standard and expressed as mg.

### 2.13 Statistical Analysis

The data were statistically analyzed using the STATISTICA software system, version 8.0 (Stat Soft. Inc., Tulsa, OK, USA). Significant differences were assessed using Student’s *t*-test and one-way or two-way analysis of variance (ANOVA), depending on the experimental design. Following significant ANOVAs, multiple comparisons were performed using the Tukey HSD post hoc test. Results were expressed as mean ± standard error mean (SEM), and the significance level used in all experiments was *p* < 0.05. Graphs were generated using GraphPad Prism software (GraphPad Software, San Diego, CA, USA).

## 3 Results

### 3.1 PQ and MB Cytotoxicity in Neuroblastoma SH-SY5Y Cells

**Figure 1:**
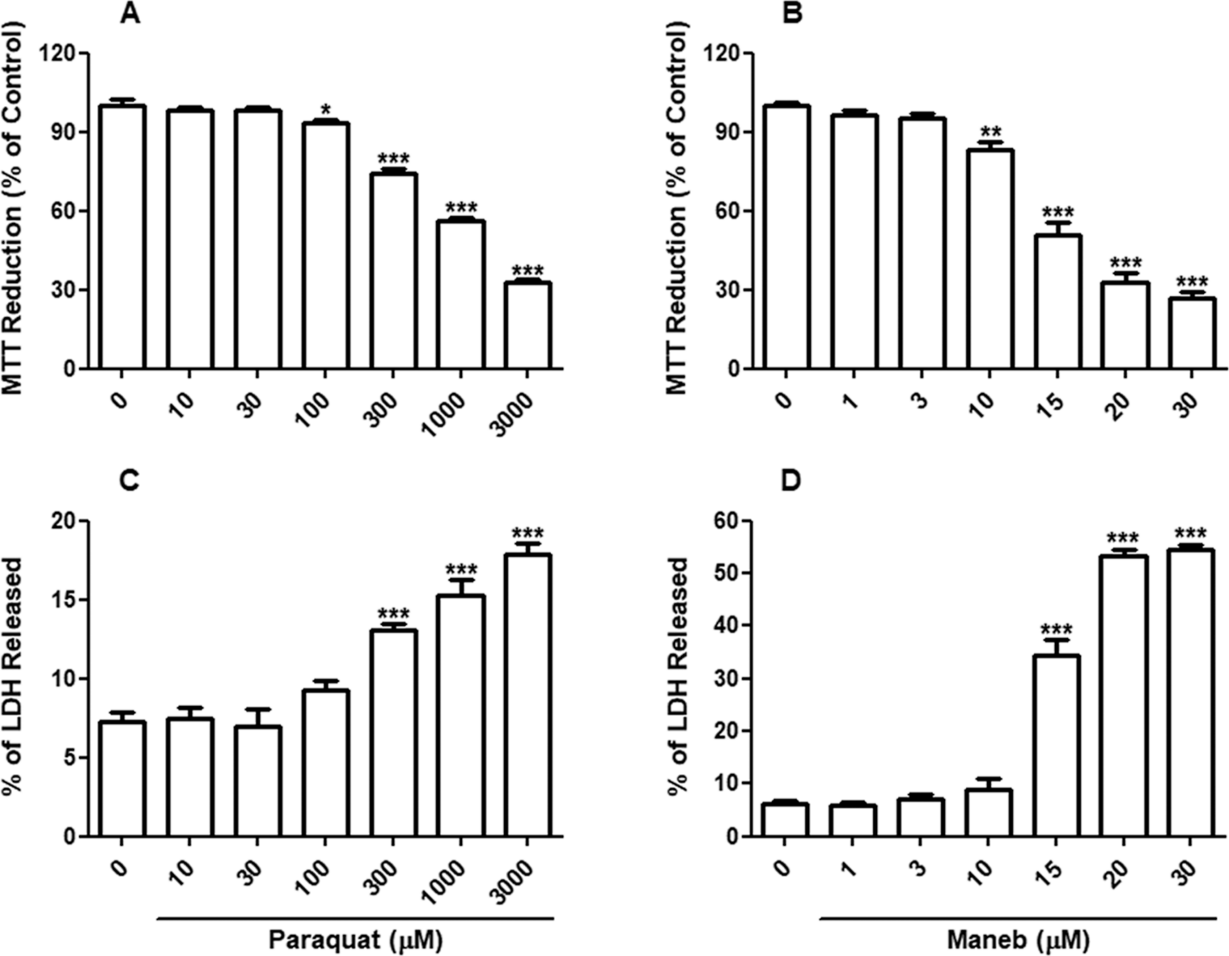
PQ and MB cytotoxicity in neuroblastoma SH-SY5Y cells. SH-SY5Y cells were incubated with vehicle (PBS or DMSO), PQ (10–3000 μM), or with MB (1–30 μM) for 24 h. Cell viability was evaluated by the reduction of MTT (A and B) and by the LDH release assay (C and D). Results of MTT assays are expressed as the percentage of MTT reduction with respect to control values. Results of LDH release assays were expressed as percent of LDH released, where the 100% value represents control cells treated with 2% Triton X-100 for 15 min. Data are represented as mean ± SEM (*n* = 8). *p < 0.05, *p* < 0.01, and ***p < 0.001 indicate statistical difference from control by one-way ANOVA, followed by Tukey’s HSD posthoc test.

Initially, we conducted concentration–response studies to assess the effects of varying concentrations of PQ (10–3000 μM) and MB (1–30 μM) on the viability of SH-SY5Y cells after 24 h of exposure. PQ and MB caused concentration-dependent declines in the cells’ capability to metabolize the MTT to formazan, with significant effects observed at 100 and 10 μM, respectively (Figure 1A,B). Furthermore, exposure to 300 μM PQ and 15 μM MB resulted in a significant disruption of the cell plasma membrane, as indicated by the increased release of LDH (*p* < 0.001, Figure 1C,D). The temporal effect of PQ and MB on SH-SY5Y cells was also investigated at different time points (12–72 h). PQ (100 μM) and MB (10 μM) exposures significantly decreased the MTT reductive capacity from 24 h and induced cytotoxicity in 48 and 72 h, as assessed through LDH leakage (Supporting Information Figure S1).

**Figure 2:**
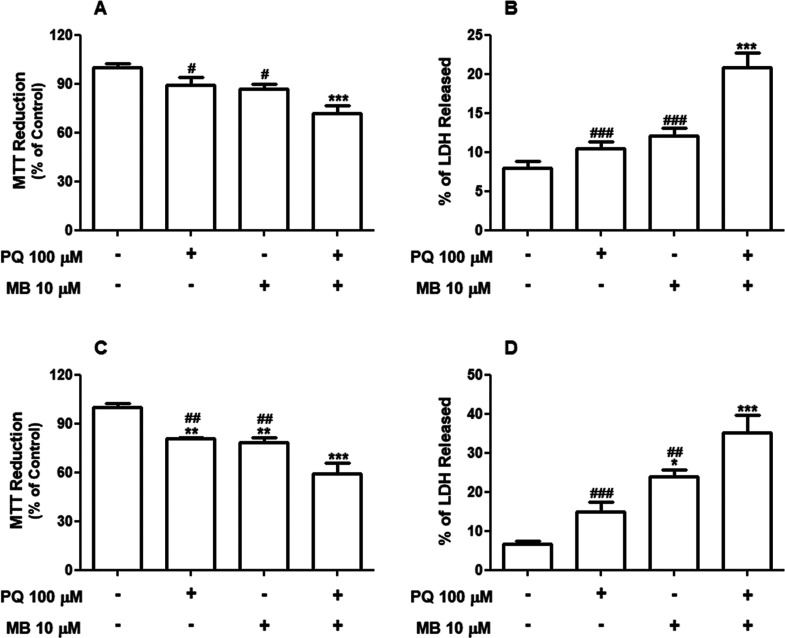
Combined exposure to low concentrations of PQ and MB induces cytotoxicity in neuroblastoma SH-SY5Y cells. Cells were exposed to 100 μM PQ and 10 μM MB alone or in combination (PQ + MB) for 24 (A and B) and 48 h (C and D). Cell viability was evaluated by the reduction of MTT (A and C) and by the LDH release assay (B and D). Results of MTT assays are expressed as the percentage of MTT reduction with respect to control values (*n* = 5–6). Results of LDH release assays were expressed as percent of LDH released, where the 100% value represents control cells treated with 2% Triton X-100 for 15 min (*n* = 5–7). Data are represented as mean ± SEM *p < 0.05, **p < 0.01, and ***p < 0.001 indicate statistical difference from control; #*p* < 0.05, ##*p* < 0.01, and ###*p* < 0.001 indicate statistical difference from PQ + MB by two-way ANOVA, followed by Tukey’s HSD posthoc test.

Next, to explore how the exposure to PQ and MB (PQ + MB) in association affects cell viability, SH-SY5Y cells were treated with 100 μM PQ plus 10 μM MB. Such concentrations (100 μM PQ plus 10 μM) were chosen because they slightly decreased the metabolic capacity to reduce MTT and did not affect the cell membrane integrity in individual exposures. Exposure to PQ and MB isolated or in combination, for 6 and 12 h (Supporting Information Figure S2), did not induce any alteration in cell viability. However, at 24 h, PQ + MB caused a significant decrease in the capacity of the cells to reduce the MTT (*p* < 0.001, Figure 2A). At this time point, PQ + MB exposure also caused a significant increase in LDH release, indicating loss of cell plasmatic membrane integrity (*p* < 0.001, Figure 2B). While post hoc analyses revealed no significant differences between control cells and those exposed to a single pesticide, a two-way ANOVA showed significant main effects of PQ on MTT reduction [*F*(1,20) = 11.31; *p* = 0.003] and LDH release [*F*(1,24) = 19.25; *p* < 0.001]. In addition, a significant main effect was observed for MB on MTT reduction [*F*(1,20) = 15.95; *p* < 0.001] and LDH release [*F*(1,24) = 43.54; *p* < 0.001]. On the other hand, no significant PQ by MB interactions were observed, neither for MTT reduction [*F*(1,20) = 0.26; *p* = 0.613] nor for LDH release [*F*(1,24) = 3.58; *p* = 0.075], clearly pointing to additive (but not synergic) effects.

Either individual or combined exposures to PQ and/or MB induced significant decreases in the MTT reductive capacity at the 48 h time point (*p* < 0.01, Figure 2C). Accordingly, the results of the two-way ANOVA demonstrated significant main effects of PQ [*F*(1,16) = 27.09; *p* < 0.001] and MB [*F*(1,16) = 35.13; *p* < 0.001] on MTT reduction. After 48 h, exposure to MB and the combination of PQ and MB significantly increased the LDH release (Figure 2D). Two-way ANOVA showed significant main effects for PQ [*F*(1,16) = 11.44; *p* = 0.003] and MB [*F*(1,16) = 30.59; *p* < 0.001] on LDH release. Again, no significant interactions between PQ and MB were observed neither on MTT reduction [*F*(1,16) = 0.00; *p* = 0.983] nor on LDH release [*F*(1,16) = 0.273; *p* = 0.607], suggesting additive effects. Photomicrographs showing the cell morphology after PQ and/or MB exposures are shown in Supporting Information Figure S3.

In summary, based on the MTT reduction assay and on the LDH release test, both PQ and MB are toxic to the neuroblastoma cells in single exposures; however, combined exposures (PQ + MB) induced a more severe cytotoxic effect, which seems to be additive and resultant from the sum of the individual effects from each pesticide.

### 3.2 PQ and MB Exposure Induces ROS Generation in SH-SY5Y Cell

**Figure 3:**
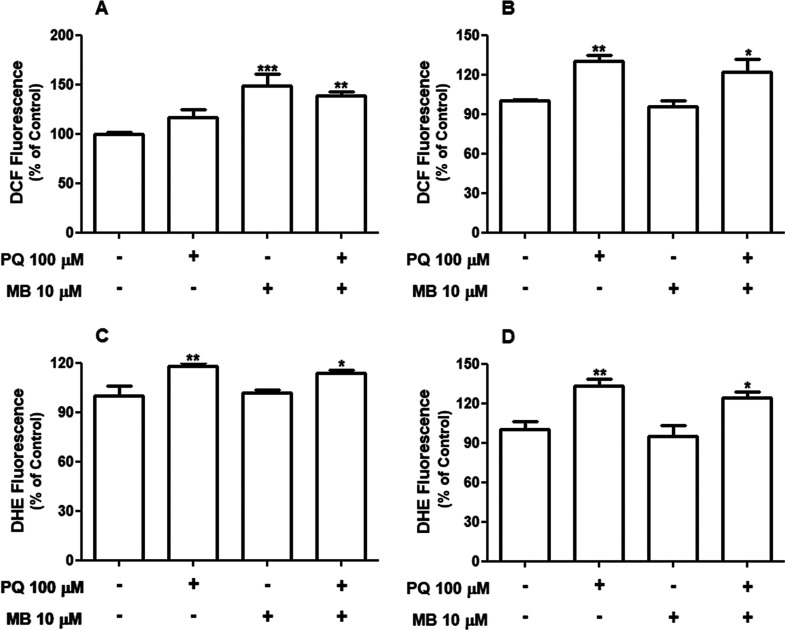
PQ and MB exposure induces reactive species production in neuroblastoma SH-SY5Y cells. Cells were incubated with vehicle (PBS or DMSO) or with 100 μM PQ and 10 μM MB alone or in combination (PQ + MB) for 6 h (A and C) or 12 h (B and D). Results of reactive species production (A and B, *n* = 8) and superoxide anion generation (C and D, *n* = 5–8) were expressed as the percentage of control. Data are represented as mean ± SEM. *p < 0.05, **p < 0.01, and ***p < 0.001 indicate statistical difference from control by two-way ANOVA, followed by Tukey’s HSD posthoc test.

Oxidative stress is a well-established mechanism underlying the toxicity induced by PQ.^7^ MB toxicity has also been associated with disruption of redox circuits.^15,17^ Thus, intracellular ROS and O_2_^•–^ production were evaluated in order to confirm the occurrence of oxidative events in PQ and/or MB exposures in SH-SY5Y cells. Both PQ and MB induced significant increases in ROS generation, however, at different time points. As shown in Figure 3A, we observed a significant increase [*F*(1,28) = 22.59; *p* < 0.001] in ROS production after MB (about 48%) or PQ + MB (about 38%) exposures at 6 h after treatments. Exposure to PQ for 6 h resulted in a slight increase (about 17%) in ROS generation; however, it was not statistically significant. On the other hand, the 12 h treatment with PQ, isolated or in association with MB, caused a statistically significant increase [*F*(1,28) = 24.22; *p* < 0.001] in ROS generation of 30% and 22%, respectively (Figure 3B).

We next evaluated O_2_^•–^ production after PQ and MB exposures. Exposure to PQ or PQ + MB for 6 h caused significant increases [*F*(1,25) = 16.79; *p* < 0.001] in O_2_^•–^ production of 18 and 13%, respectively (Figure 3C). Similarly, at 12 h, PQ and PQ + MB treatments caused statistically significant [*F*(1,21) = 26.05; *p* < 0.001] increases in O_2_^•–^ production of 33 and 23%, respectively (Figure 3D).

### 3.3 Effects of PQ and/or MB on Mitochondrial Parameters in SH-SY5Y Cells

**Figure 4:**
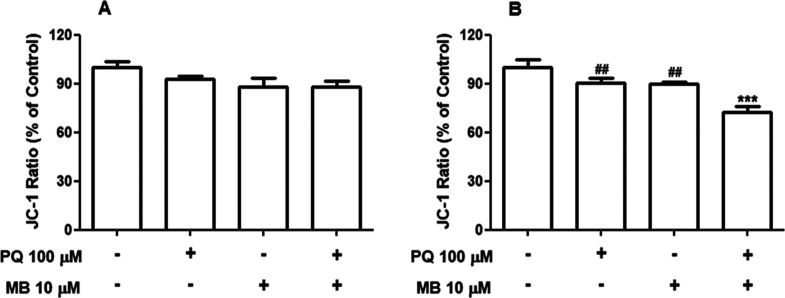
Combined exposure to low concentrations of PQ and MB decreases the MMP in neuroblastoma SH-SY5Y cells. Cells were incubated with vehicle (PBS or DMSO) or with 100 μM PQ and 10 μM MB alone or in combination (PQ + MB) for 6 (A) or 12 h (B). The MMP was determined from the ratio of fluorescence intensity from JC-1 aggregates (red) and monomeric (green) fluorescence. Results were expressed as the percentage of control. Data are represented as mean ± SEM (*n* = 5–7). ***p < 0.001 indicates statistical difference from control; ##*p* < 0.01 indicates statistical difference from PQ + MB by two-way ANOVA, followed by Tukey’s HSD posthoc test.

**Figure 5:**
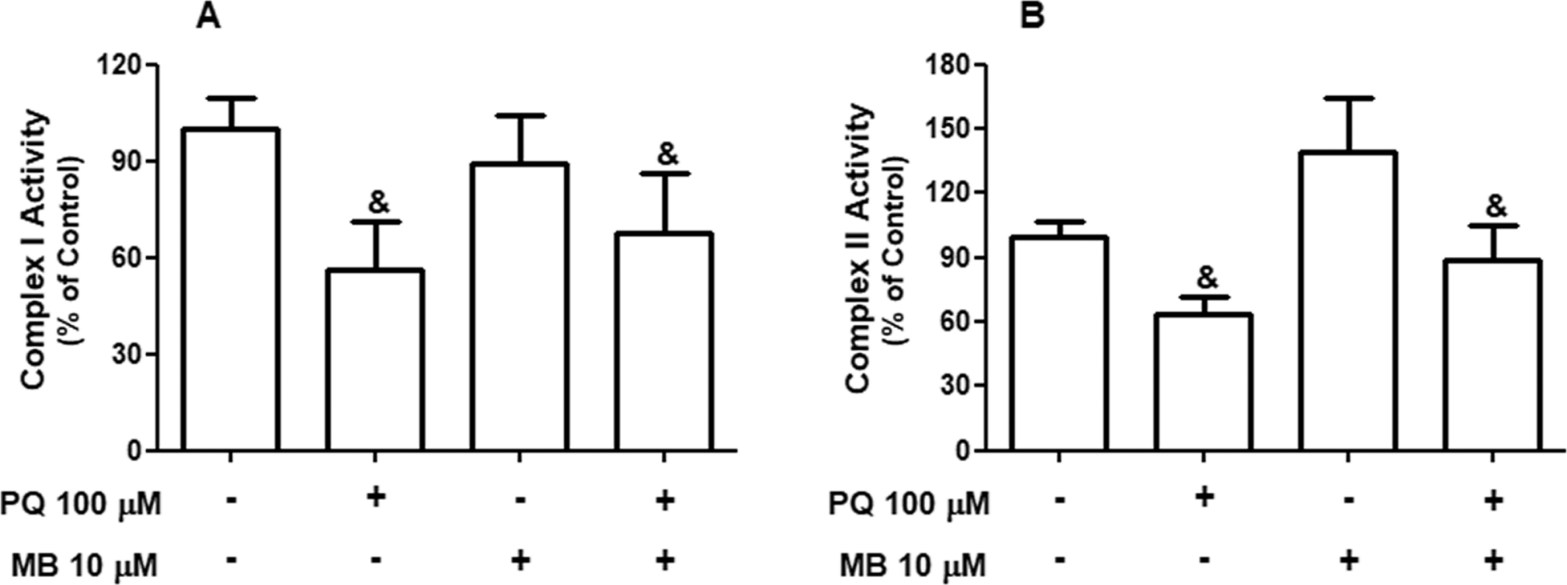
Effects of PQ and MB on mitochondrial complex I and complex II activities in neuroblastoma SH-SY5Y cells. Cells were incubated with vehicle (PBS or DMSO) or with 100 μM PQ and 10 μM MB alone or in combination (PQ + MB) for 12 h. NADH dehydrogenases (complex I) activity (A) was measured by the rate of NADH-dependent ferricyanide reduction in 340 nm and calculated as nmol/min/mg protein (*n* = 4). Complex II activity (B) was measured by following the decrease in absorbance due to the reduction of 2,6-DCIP at 600 nm, calculated as nanomole of 2,6-DCIP reduced/min/mg of protein and (*n* = 5). Results were expressed as the percentage of control. Data are represented as mean ± SEM and & indicate a significant (*p* < 0.05) main effect of PQ by two-way ANOVA, followed by Tukey’s HSD posthoc test.

To better understand the mechanisms through which PQ and MB cause toxicity in SH-SY5Y cells, we examined their effects on MMP and complex I and II activities. No significant effects of the pesticides were observed at 6 h on the MMP. However, at 12 h of exposure, PQ + MB induced a significant decrease in the MMP (*p* < 0.001, Figure 4B). Two-way ANOVA indicated significant main effects of PQ [*F*(1,16) = 15.55; *p* = 0.0011] and MB [*F*(1,16) = 17.05; *p* < 0.001] on MMP. However, there were no significant interactions between PQ and MB on MMP [*F*(1,16) = 1.30; *p* = 0.27]. In addition, two-way ANOVA showed a main effect of PQ on mitochondrial complex I [*F*(1,12) = 4.81; *p* = 0.048, Figure 5A] and on complex II [*F*(1,16) = 7.38; *p* = 0.0152, Figure 5B] activities. On the other hand, MB did not cause any significant alterations in the activities of mitochondrial complexes I and II.

### 3.4 Effects of PQ and/or MB on GSH Levels in SH-SY5Y Cells

**Figure 6:**
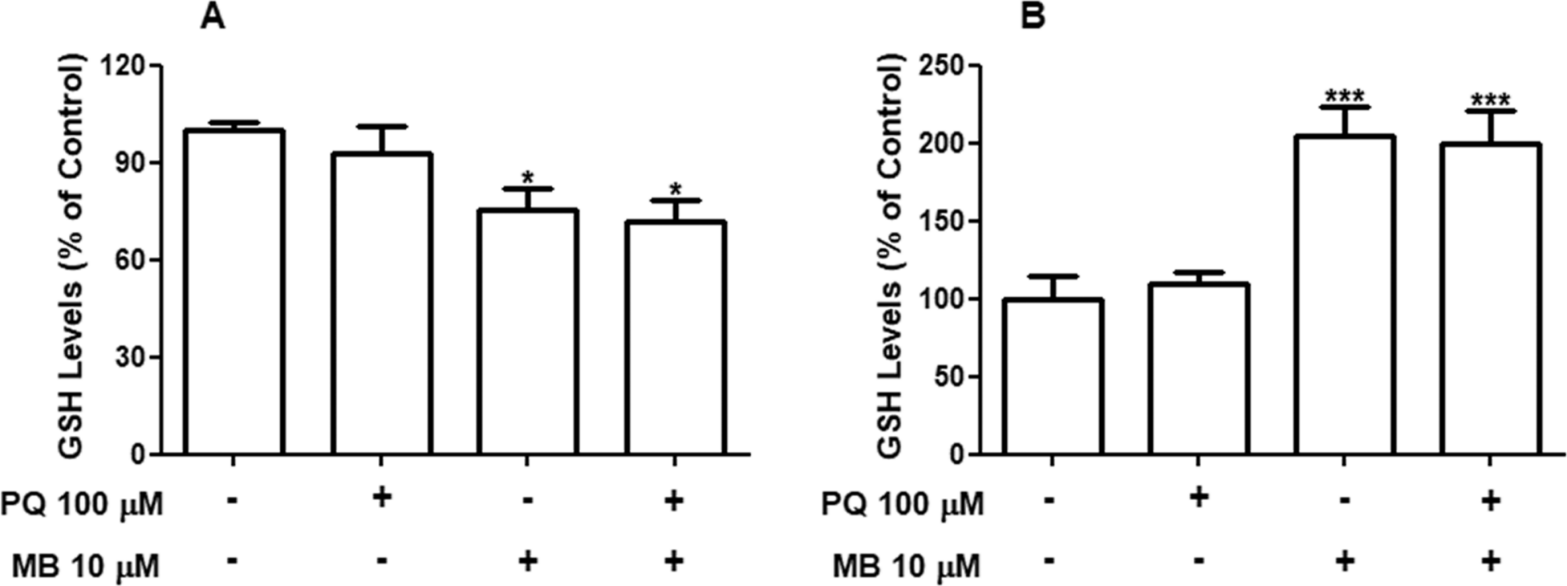
Effects of PQ and MB on GSH levels in neuroblastoma SH-SY5Y cells. Cells were incubated with vehicle (PBS or DMSO) or with 100 μM PQ and 10 μM MB alone or in combination (PQ + MB) for 6 h (A) and 12 h (B). GSH levels were determined as nonprotein thiols (NPSH), whose levels in the control condition were 6.46 ± 0.55 nmol of NPSH/mg protein. Results were expressed as the percentage of control. Data are represented as mean ± SEM (*n* = 6–7). *p < 0.05 and ***p < 0.001 indicate statistical difference from control by two-way ANOVA, followed by Tukey’s HSD posthoc test.

**Figure 7:**
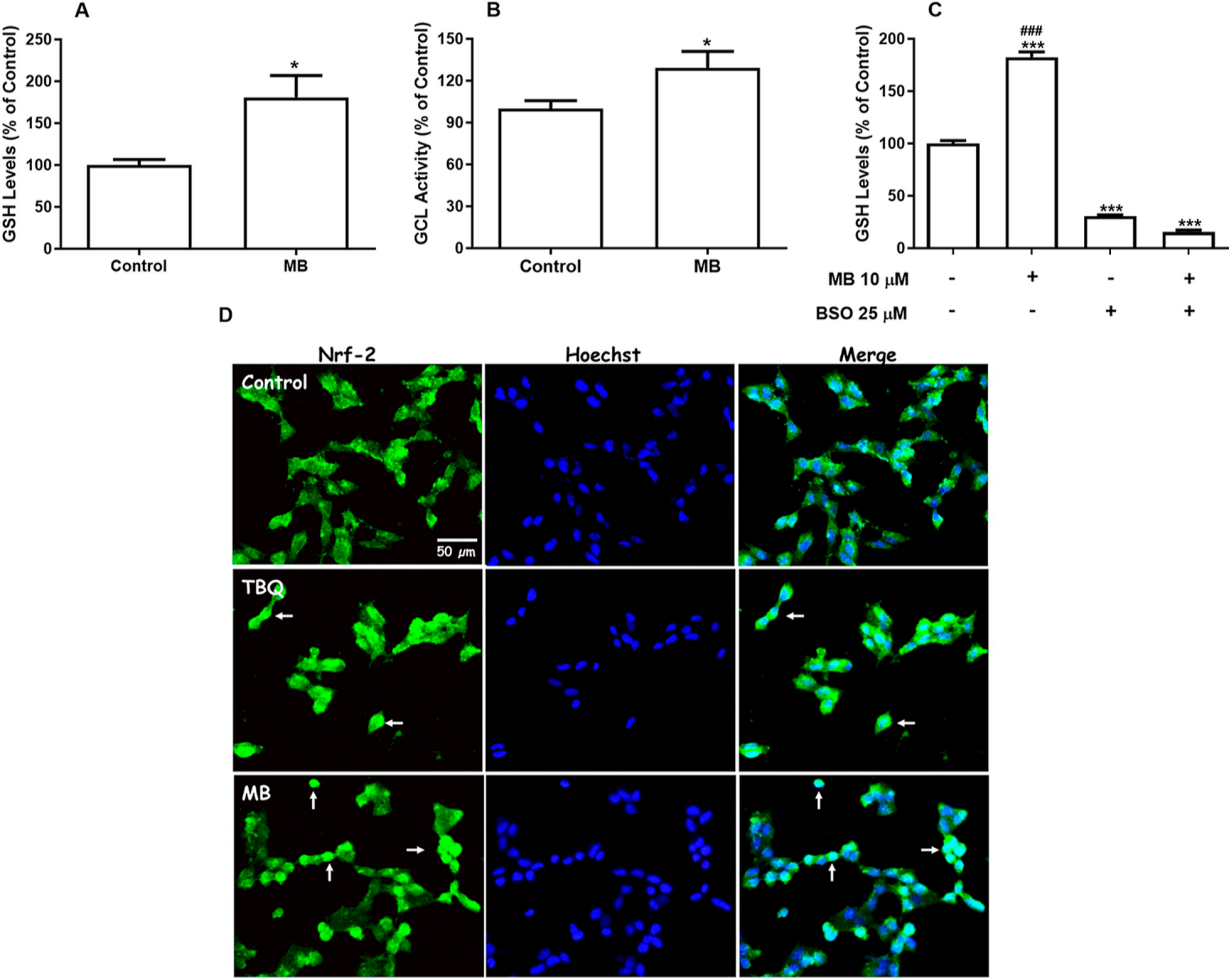
Effects of MB on GSH levels, GCL activity, and Nrf2 nuclear translocation in neuroblastoma SH-SY5Y cells. Cells were incubated with vehicle (DMSO) or with 10 μM MB for 12 h (A and B). GSH levels were determined as nmol GSH/mg of protein, and the results were expressed as percentage of control (*n* = 4). GCL activity was expressed as a percentage of control, whose activity was 6.77 ± 0.78 nmol of NADH oxidized/min/mg protein (*n* = 4). Data are represented as mean ± SEM *p < 0.05 indicates statistical difference from control using the Student’s *t*-test (A and B). Cells were incubated with 25 μM BSO for 10 h and then were exposed to 10 μM MB for 12 h (C). GSH levels were determined as nonprotein thiols (NPSH), and the results were expressed as the percentage of control (*n* = 4). Data are represented as mean ± SEM. ***p < 0.001 indicates statistical difference from control; ###*p* < 0.001 indicates statistical difference from MB + BSO by two-way ANOVA, followed by Tukey’s HSD posthoc test. Cells were incubated with vehicle, with 10 μM TBQ (positive control), or with MB for 6 h (D). The nuclear translocation of Nrf2 was evaluated by immunofluorescence (green). Cell nuclei were stained with Hoechst 33258 (blue). A representative merge of the green and blue fluorescence is shown. (Magnification 200×).

PQ and MB exposure alter the ROS generation in SH-SY5Y cells and disrupts the MMP when the pesticides are combined. To better understand the role of oxidative stress in PQ and/or MB-induced toxicity, cellular GSH levels were evaluated as nonprotein thiol. As shown in Figure 6A, we found a significant decrease (about 27%) [*F*(1,24) = 13.41; *p* = 0.0012] in GSH levels after MB or PQ + MB exposures for 6 h. Notably, at 12 h, MB significantly increased the cellular content of GSH (approximately 100%), independently of PQ treatment [*F*(1,20) = 50.92; *p* < 0.001]. Conversely, PQ alone did not significantly alter the GSH content at any evaluated time point (Figure 6). To confirm the increase in GSH levels by MB, we have performed the measurement of GSH content by another methodology which is more specific for GSH content measurement. As shown in Figure 7A, MB significantly increased the GSH levels after 12 h of exposure, confirming the result in Figure 6B.

To understand the mechanisms behind the MB-induced increase in the level of GSH in SH-SY5Y cells, we measured the GCL activity. GCL catalyzes the initial and crucial step in the GSH synthesis pathway.^40^ As shown in Figure 7, MB significantly increased the GCL activity at 12 h of exposure (*p* < 0.05, Figure 7B). In order to confirm the involvement of GCL on the increase of GSH levels in MB-treated cells, experiments were performed with BSO, a specific GCL inhibitor.^41^ BSO decreased approximately 70% the cellular content of GSH (*p* < 0.001, Figure 7C). The same inhibitory effect was observed when cells were treated with MB in association with BSO, indicating that BSO abolishes the MB-mediated GSH increase. These findings indicate that the rise in cellular GSH levels observed after exposure to MB is caused by enhanced GSH synthesis through the up-regulation of GCL.

GCL gene expression is known to increase through activation of the Nrf2/antioxidant response element (ARE) pathway.^40^ In order to understand how MB might enhance GCL activity and GSH levels, we explored whether MB could promote Nrf2 nuclear translocation in SH-SY5Y cells. Exposure to MB for 6 h increased Nrf2 staining in cell nuclei, similarly to cells treated with the positive control TBQ (Figure 7D). This result indicates that the increase in GSH after MB exposure seems to be linked to the activation of the Nrf2/ARE pathway.

**Figure 8:**
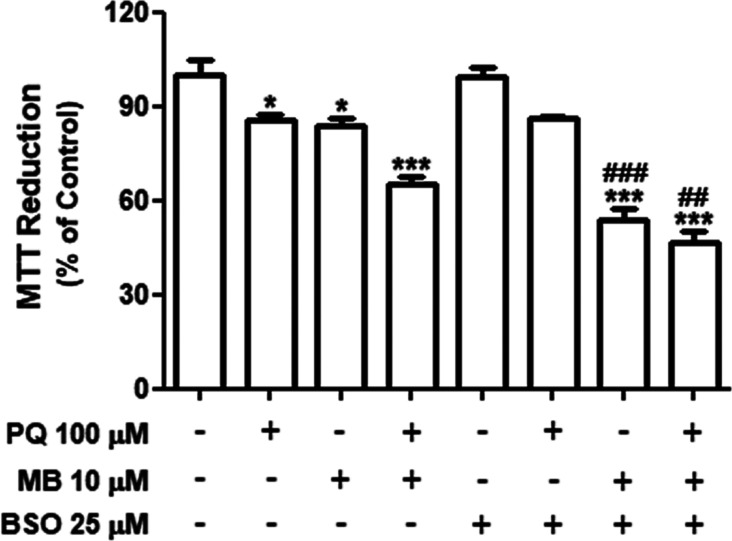
Effects of PQ and/or MB and BSO on cell viability in neuroblastoma SH-SY5Y cells. Cells were incubated with BSO (25 μM) for 24 h and then exposed to 100 μM PQ and 10 μM MB alone or in combination (PQ + MB) for 48 h. Cell viability was evaluated by the reduction of MTT, and the results are expressed as the percentage of MTT reduction with respect to control values. Data are represented as mean ± SEM (*n* = 7). *p < 0.05 and ***p < 0.001 indicate statistical difference from control; ##*p* < 0.01 and ###*p* < 0.001 indicate statistical difference from PQ + MB and from MB, respectively, by two-way ANOVA, followed by Tukey’s HSD posthoc test.

To investigate the involvement of endogenous GSH in pesticide-induced toxicity, cells were pretreated with BSO in the presence or absence of PQ and/or MB. While BSO alone did not significantly affect cell viability, the treatment of BSO in association with MB significantly enhanced the cytotoxic effect of the fungicide on SH-SY5Y cells (*p* < 0.001, Figure 8). In contrast, the cytotoxicity of PQ was not potentiated by BSO. Thus, GSH depletion (induced by BSO treatment) makes neuroblastoma cells more sensitive to MB-induced toxicity. This data indicate that GSH is an important defense system against MB-induced toxicity.

### 3.5 NAC Protective Effect on PQ and/or MB-Induced Toxicity in SH-SY5Y Cells

**Figure 9:**
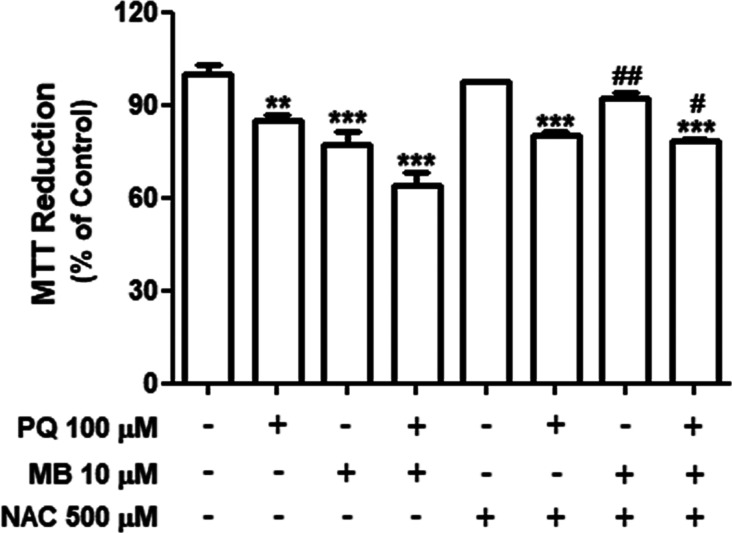
NAC treatment protects from MB-induced cytotoxicity in neuroblastoma SH-SY5Y cells. Cells were pretreated with NAC (500 μM) for 1 h and then exposed to 100 μM PQ and 10 μM MB alone or in combination (PQ + MB) for 48 h. Cell viability was evaluated by the reduction of MTT, and the results are expressed as the percentage of MTT reduction with respect to control values. Data are represented as mean ± SEM (*n* = 3). **p < 0.01 and ***p < 0.001 indicate statistical difference from control; #*p* < 0.05 and ##*p* < 0.01 indicate statistical difference from PQ + MB and from MB, respectively, by two-way ANOVA, followed by Tukey’s HSD posthoc test.

In order to explore the contribution of GSH to PQ and/or MB-induced toxicity, neuroblastoma cells were pretreated with *N*-acetylcysteine, an antioxidant compound, 1 h prior pesticide exposure. NAC pretreatment significantly protected from MB-induced toxicity [*F*(1,16) = 25.25; *p* < 0.001] (Figure 9). On the other hand, NAC pretreatment failed to protect from PQ-induced decrease in cell viability and displayed only a partial protective effect against PQ + MB-induced toxicity at 48 h after pesticide exposure. However, the decrease in cell viability induced by PQ + MB at 24 h was efficiently protected by NAC treatment (Supporting Information Figure S4).

## 4 Discussion

Epidemiological evidence has revealed that the combined exposure to the pesticides PQ and MB raises the risk of developing PD in humans.^4^ In rodents, the administration of PQ and MB resembles many key aspects of PD, such as motor impairments and the loss of dopaminergic neurons in the SN.^8,9,11^ In combined exposure scenarios, PQ and MB produce more pronounced toxic effects compared to treatments with each toxicant alone.^16,20^ However, the mechanisms behind PQ + MB-induced toxicity are not fully understood. Therefore, we studied the cytotoxic effects induced by the pesticides PQ and MB in combination (PQ + MB) in SH-SY5Y cells. The results presented here demonstrate that the cytotoxic effect of the pesticides was increased in combined exposures (PQ + MB), which may represent additive toxic consequences resulting from PQ and MB exposures. This is supported by the fact that a two-way ANOVA indicated no significant PQ by MB interactions for MTT reduction, LDH release, and MMP (JC-1 assay), pointing to additive (but not synergic) effects.

Simultaneous exposure to different agrochemicals is a widespread phenomenon worldwide. This highlights increasing concerns regarding the possible detrimental effects of exposures to mixtures of compounds. In general, both research toxicity studies and pesticide registration protocols focus on assessing the effects of isolated active substances, while the effects of combined compounds are only assessed when those are part of the same formulation. However, the simultaneous application of two or more pesticides in the same agricultural area is a common event during the same cropping season.^22,23^ Hence, a complex cocktail of agrochemicals, which are not coformulated as single products, can be simultaneously detected in food and water samples.^42−44^

The toxic effects of mixtures of pesticides have been investigated in the past several years. As already mentioned, epidemiologic evidence indicates that combined exposure to PQ and MB enhances the risk for PD developing in human populations.^4^ In line with this, experimental studies have shown more severe toxic effects of combined exposure to PQ and MB on the dopaminergic system of rodents.^16,20^ In our study, we demonstrated that exposure to PQ and MB in combination was more toxic to SH-SY5Y cells than exposure to a single pesticide. In single exposures, both pesticides decreased the cell viability. However, when cells were exposed to a mixture of both pesticides, at low concentrations, we observed a significant decrease in cell viability and cell membrane disruption. These findings suggest that the changes in cell viability are more likely due to additive toxic events rather than synergistic toxic effects from PQ and MB exposures. This idea is reinforced by the absence of significant interactions between PQ and MB in both MTT reduction and LDH release assays.

In fact, our data indicate that both pesticides seem to induce toxicity by divergent mechanisms at different time points. Both PQ and MB are able to increase the production of reactive species at different time points, but these effects were not potentiated by each other. PQ significantly increased the production of ROS at 12 h of exposure, which mostly represents superoxide anion generation. This was confirmed by the fact that similar increases were observed in the ROS (about 17 and 30%) and O_2_^•–^ generation (about 18 and 33%) at 6 and 12 h of exposure, respectively. It is well described that the primary toxic effect of PQ is linked to its capacity to initiate a redox cycle, leading to the production of O_2_^•–^.^7^ Although several studies suggest that O_2_^•–^ is not a very reactive radical in biological systems, it can act as a precursor for the generation of extremely reactive and dangerous species, including hydroxyl radicals (^•^OH) and peroxynitrite (ONOO^–^).^45,46^ Both ^•^OH and ONOO^–^ can promote oxidation of biological components such as lipids, proteins, and DNA, leading to cell death.^47^ Thus, an increase of superoxide, directly or indirectly, has deleterious effects that may culminate in oxidative stress. In this scenario, antioxidant systems are essential to controlling the levels of these reactive species. Superoxide dismutase (SOD) can enzymatically dismutase O_2_^•–^ in H_2_O_2_, which then is reduced to H_2_O by the catalase and GSH peroxidase (GPx) system.^48^ SOD mimetics were neuroprotective against PQ-induced toxicity,^49^ highlighting an important role of superoxide anion generation and oxidative stress in the mechanism of PQ-mediated toxicity.

Although PQ increases ROS and O_2_^•–^ generation in SH-SY5Y cells, no alteration was observed in GSH levels. Our findings align with a previous study indicating that exposure to 100 μM PQ for 24 h was not able to deplete cellular GSH content in neuroblastoma cells.^17^ Thus, despite a significant increase in the generation of ROS, GSH levels were maintained after PQ exposure, indicating that in this condition the detoxification of ROS generated by PQ takes place without GSH depletion or, alternatively, that the GSH turnover is enough to maintain its levels under basal values.

On the other hand, MB was also able to increase ROS production; however, this event was observed at 6 h after exposure and without any alteration in O_2_^•–^ generation. There are few data suggesting that MB induces ROS formation in tissues. Previous studies showed that, in contrast to PQ, MB is not able to induce ROS formation.^17^ However, in a study by Roede and colleagues, the authors observed an increased ROS production at 1 h after MB exposure. In our study, MB caused an increase in ROS formation only at 6 h but not at 12 h of MB exposure. The increase in ROS generation induced by MB was not accompanied by an increase in O_2_^•–^ production, indicating that other reactive species could be produced after MB exposure. We used the probe DCFH-DA to detect ROS formation. DCFH-DA is extensively employed as a fluorescent probe for ROS detection, especially H_2_O_2_. However, its use in ROS detection is debated in the literature, with evidence suggesting that this probe can detect nitrogen reactive species as well.^50,51^ Thus, additional detailed studies are necessary to address the possible mechanism of the MB-inducing ROS generation.

In our study, at 6 h after MB exposure, the observed increase in ROS production was paralleled by a decrease in the cellular content of GSH. The decrease in GSH content at 6 h after MB exposure may be related to the increased production of ROS. Our data are in accordance with a previous study showing that MB exposure for 2 h affected the cellular thiol redox status of SK-N-AS human neuroblastoma cells. This effect involved the oxidation of cellular GSH and changes in the thiol redox status of the enzyme peroxiredoxin 3.^52^

However, at 12 h of MB exposure, we observed the opposite effect. At this time point, the DCF fluorescence of cells exposed to MB returned to control levels and, at the same time, MB induced a dramatic increase in cellular GSH content, an effect already described in primary mesencephalic cultures and in PC12 cells^11^ and in SH-SY5Y cells.^17^ The increase in GSH content was attributed to an increase in GSH synthesis.^17^ In our study, MB exposure was able to increase GCL activity, the rate-limiting enzyme in the GSH synthesis, an effect previously observed in PC12 cells.^11^ In addition, the incubation of neuroblastoma cells with BSO, a classical GCL inhibitor, abolished the increase in GSH levels induced by MB, corroborating with previous studies.^11,17^

The tripeptide GSH is one of the most important thiol antioxidants and redox buffers of the cells. GSH acts independently or in conjunction with other enzymes to neutralize oxidants, providing protection against oxidative stress-induced damage^53,54^ GSH is produced in the cytosol through the sequential actions of GCL and GSH synthetase. GCL, the rate-limiting enzyme in GSH synthesis, is known to be upregulated via the activation (nuclear translocation) of the Nrf2/ARE pathway.^40^ Nrf2 is a transcription factor that controls the expression of various phase II antioxidant genes, such as GSH S-transferase, heme oxygenase 1, and the thioredoxin and peroxiredoxin systems, along with numerous enzymes involved in GSH metabolism. Under normal conditions, Nrf2 is retained in the cytosol by Keap-1, a protein that negatively regulates Nrf2. Under situations of oxidative stress, occurs the dissociation of Nrf2 from Keap1 and its translocation to the nucleus. In the nucleus, Nrf2 attaches to ARE, enhancing the transcription of antioxidant and cytoprotective genes.^55^ In the current study, MB was capable of inducing nuclear translocation of Nrf2 after 6 h of exposure (Figure 7D), indicating that the substantial increases in GCL activity and GSH content observed at 12 h after MB exposure are related to the activation of the Nrf2 pathway in MB-treated SH-SY5Y cells. Our data are in accordance with Roede and co-workers who have shown that MB is able to increase Nrf2 translocation to the nucleus and upregulates the mRNA for phase II detoxification enzymes regulated by Nrf2.^17^

Even though MB increases GSH levels at 12 h, this event did not prevent a decrease in cell viability after 48 h of MB exposure. However, it may mitigate, at least in part, the toxic effect of MB on cell viability. This is supported by the fact that the cytotoxicity of MB was increased in neuroblastoma cells pretreated with BSO (Figure 8). These data show that GSH depletion increases the vulnerability of neuroblastoma cells to MB-induced toxicity. Additionally, the treatment with NAC, protected against MB-induced toxicity, indicating that GSH is an important defense system against MB cytotoxicity. NAC is an antioxidant molecule that can act as a free radical-scavenging compound and, in addition, is a source of cysteine for GSH synthesis.^56,57^ With respect to PQ, the GSH depletion by BSO treatment did not potentiate PQ-induced toxicity. In addition, NAC treatment failed to prevent the decrease in cell viability induced by PQ and provided only partial protection against PQ + MB exposure after 48 h. On the other hand, NAC was able to protect from PQ + MB-induced decrease in cell viability at 24 h of exposure. These data indicate that, despite the established role of oxidative stress as a primary mechanism of PQ-induced toxicity, other mechanisms may also be contributing to the PQ toxic effect in SH-SY5Y cells at 48 h of exposure, and the treatment with NAC is not effective in reducing PQ toxicity. Furthermore, besides oxidative stress appearing as a mechanism of toxicity of both pesticides, the partial protective effect of NAC on PQ + MB- induced reduction in cell viability highlights that the cytotoxic effect of the combined exposure to the pesticides at 48 h represents additive consequences of both compounds acting by different pathways.

To further our understanding of the mechanisms underlying PQ and MB-induced toxicity, we also explored how these pesticides disrupt mitochondrial function in SH-SY5Y cells. The exposure to PQ caused a decrease in the activities of both complex I and complex II. In contrast, MB exposure did not affect complex I or II activities. However, exposure to MB in isolated rat brain mitochondria showed a preferential suppression of mitochondrial complex III.^12^ Additionally, single exposures to the pesticides did not change the MMP. In previous studies in SH-SY5Y cells, exposure to 100 μM PQ for 24 h resulted in a significant reduction of approximately 50% in complex I activity, which was accompanied by a reduction of approximately 50% in the MMP.^58,59^ Our data show that PQ exposure for 12 h decreased complex I and II activities, and this effect was not statistically significant from control cells. However, two-way ANOVA revealed a significant main effect of PQ on both mitochondrial complex I [*F*(1,12) = 4.81; *p* = 0.048, Figure 5A] and on complex II [*F*(1,16) = 7.38; *p* = 0.0152, Figure 5B] activities, indicating that the herbicide changes these parameters. Even with the main effect of PQ (100 μM, 12 h of exposure) on the complex I and II activities, the decrease in the activity of these complexes was not enough to promote a strong decrease in the MMP. Thus, the minor effect of PQ on MMP in our study may be related to a minor effect on mitochondrial complex I and II activities after 12 h of exposure.

On the other hand, combined exposure to PQ + MB for 12 h decreased the MMP. Our findings align with previous studies indicating that the combination of PQ and MB reduces the MMP in dopamine-like neurons and in human nerve-like cells.^18,60^ The MMP has an essential role in maintaining cellular homeostasis, and a drop of MMP may induce loss of cell viability and apoptosis.^61^ The toxicity of several xenobiotic compounds can have either a primary or a secondary effect on mitochondrial function. Many of these compounds reduce MMP by acting on a variety of different targets in the mitochondria and therefore affecting the mitochondrial function.^62,63^

Both PQ and MB can affect mitochondrial function. Previous studies have demonstrated that mitochondria are a primary source of ROS production induced by PQ.^64,65^ Overexpression of manganese superoxide dismutase (MnSOD), the mitochondrial isoform of SOD, inhibited oxidative stress and cell death.^66^ On the other hand, suppression of MnSOD enhances sensitivity to PQ-induced toxicity.^67^ Moreover, a recent study showed that PQ cytotoxicity through redox cycling might result in impairing mitochondrial membrane permeability.^68^ Together, these evidence, demonstrate a pivotal role of mitochondrial ROS production in PQ-mediated toxicity.^66,67^ It has been described that multiple mitochondrial sites, notably, complex I,^65^ and complex III,^64,69^ contribute to ROS generation after mitochondrial incubation with PQ. Although Richardson and colleagues have shown that PQ (even at millimolar concentration) seems not to be an effective inhibitor of complex I in isolated brain mitochondria,^70^ several studies have demonstrated a decrease in complex I activity after PQ exposures.^71,72^ Our study demonstrated a reduction in complex I and II activities following 12 h of PQ exposure. Although controversial, these data contribute to explaining the toxicity of PQ to mitochondria. Through the generation of mitochondrial ROS, PQ can indirectly impair mitochondrial function, thereby contributing to cellular damage. In comparison to PQ, there are fewer studies regarding the effects of MB on mitochondria. As mentioned above, MB was already shown to inhibit mitochondrial complex III.^12^ In addition, sub cytotoxic exposure to MB decreases mitochondrial oxygen consumption and disrupts various cellular energy pathways, resulting in a significant decrease in ATP synthesis in human neuroblastoma cells.^13^ By different mechanisms, both pesticides seem to play a role in MMP disruption in combined exposures. In fact, the combined exposure to PQ + MB decreases the MMP, probably by the additive effects of both pesticides. This was reinforced by the fact that no significant interaction was observed in the MMP after PQ + MB exposure.

## 5 Conclusions

In summary, the findings presented here show that PQ + MB cytotoxicity to SH-SY5Y cells does not occur via synergistic or potentiation mechanisms. Instead, our findings indicate that the cytotoxic effect induced by the simultaneous exposures to PQ and MB is a result of the additive effects of both pesticides acting through oxidative events at different time points. PQ induces ROS production, particularly O_2_^•–^ affecting cellular mitochondrial function. MB also induces ROS production with depletion of GSH content at short time points of exposure followed by GCL upregulation and increased GSH levels, which may be related to Nrf2/Are pathway activation. Together, these mechanisms lead to the disruption of the MMP, compromising cell viability. Thus, our study contributes to a better understanding of the cytotoxicity of PQ and MB in combined exposures. The investigation of potential effects of the combination of pesticides has an essential role in contributing to the understanding of detrimental effects of mixtures to human health and the environment. Due to current regulatory assessments focusing solely on individual pesticides, there is an urgent need for more studies on the toxicity of pesticide mixtures.

## Supporting Information Available

MTT reduction, LDH release, and photographs of cells (PDF)

## Abbreviations

BSO: buthionine sulfoximine
DMSO: dimethyl sulfoxide
GCL: glutamate cysteine ligase
GSH: glutathione
LBs: Lewy bodies
LDH: lactate dehydrogenase
MB: maneb
MMP: mitochondrial membrane potential
PQ: paraquat
NAC: *N*-acetylcysteine
PD: Parkinson’s disease
RS: reactive species
SNpc: substantia nigra pars compacta
