# Supplementary material for: Mechanisms Mediating the Combined Toxicity of Paraquat and Maneb in SH-SY5Y Neuroblastoma Cells
Source: Chem Res Toxicol. 2024 Jul 26;37(8):1269–82. doi: 10.1021/acs.chemrestox.3c00389 (PMC11337211; doi:10.1021/acs.chemrestox.3c00389)
Supplement: Supplementary file 1 — tx3c00389_si_001.pdf [file tx3c00389_si_001.pdf]

Supporting Information

Mechanisms mediating the combined toxicity of Paraquat and Maneb in SH-SY5Y neuroblastoma cells

Suzana da Silva [a,c], Carolina de Lima da Costa [a,c], Aline Aita Naime [b], Danúbia Bonfanti Santos [b], Marcelo Farina [b,*], Dirleise Colle [a,*]

[a]Department of Clinical Analyses, Federal University of Santa Catarina, Florianopolis 88040-900, Santa Catarina, Brazil

[b]Department of Biochemistry, Federal University of Santa Catarina, Florianopolis 88040-900, Santa Catarina, Brazil

[c] These authors contributed equally

***Corresponding author:** Dirleise Colle[a] (dirleise.colle@ufsc.br; dirleise@yahoo.com.br/ ORCID: 0000-0003-2345-5297) and Marcelo Farina[b] (marcelo.farina@ufsc.br/ORCID: 0000-0001-8255-8515). [a]To whom correspondence should be eddressed at Departamento de Análises Clínicas, Centro de Ciências da Saúde, Universidade Federal de Santa Catarina, CEP 88040-900, Florianópolis, Santa Catarina, Brazil. Phone: +55 4837213472. [b]Departamento de Bioquímica, Centro de Ciências Biológicas, Bloco C, Campus Universitário Trindade, Universidade Federal de Santa Catarina, CEP 88040-900, Florianópolis, Santa Catarina, Brazil. Phone: +55 4837214580.

**Content**	**Page**
**MTT reduction and LDH release**	**S2**
**MTT reduction and LDH release**	**S3**
**Cells images**	**S4**
**MTT reduction**	**S5**

Figure S1. PQ and MB cytotoxicity in neuroblastoma SH-SY5Y cells at different time-points.

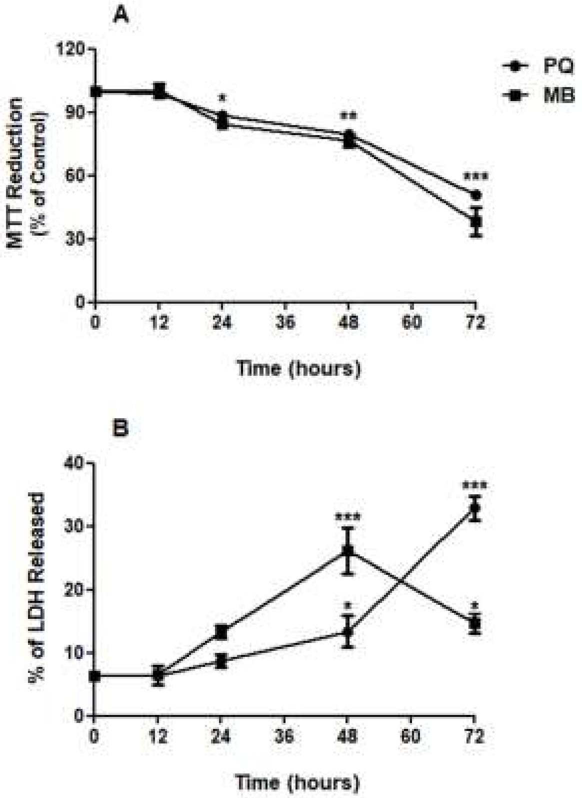

Legend. SH-SY5Y cells were incubated with vehicle (PBS or DMSO), PQ (100 μM) or with MB (10 μM) for 12, 24, 48 and 72 h. Cell viability was evaluated by the reduction of MTT (A) and by the LDH release assay (B). Results of MTT assays are expressed as the percentage of MTT reduction with respect to control values. Results of LDH release assays were expressed as percent of LDH released, where the 100% value represents control cells treated with 2% Triton X-100 for 15 min. Data are represented as mean ± S.E.M. (n= 6). * $p < 0.05$, $p < 0.01$ and *** $p<0.001$ indicate statistical difference from control by one-way ANOVA, followed by Tukey HSD post-hoc test.

Figure S2. Effects of combined exposure to low concentrations of PQ and MB in neuroblastoma SH-SY5Y cells.

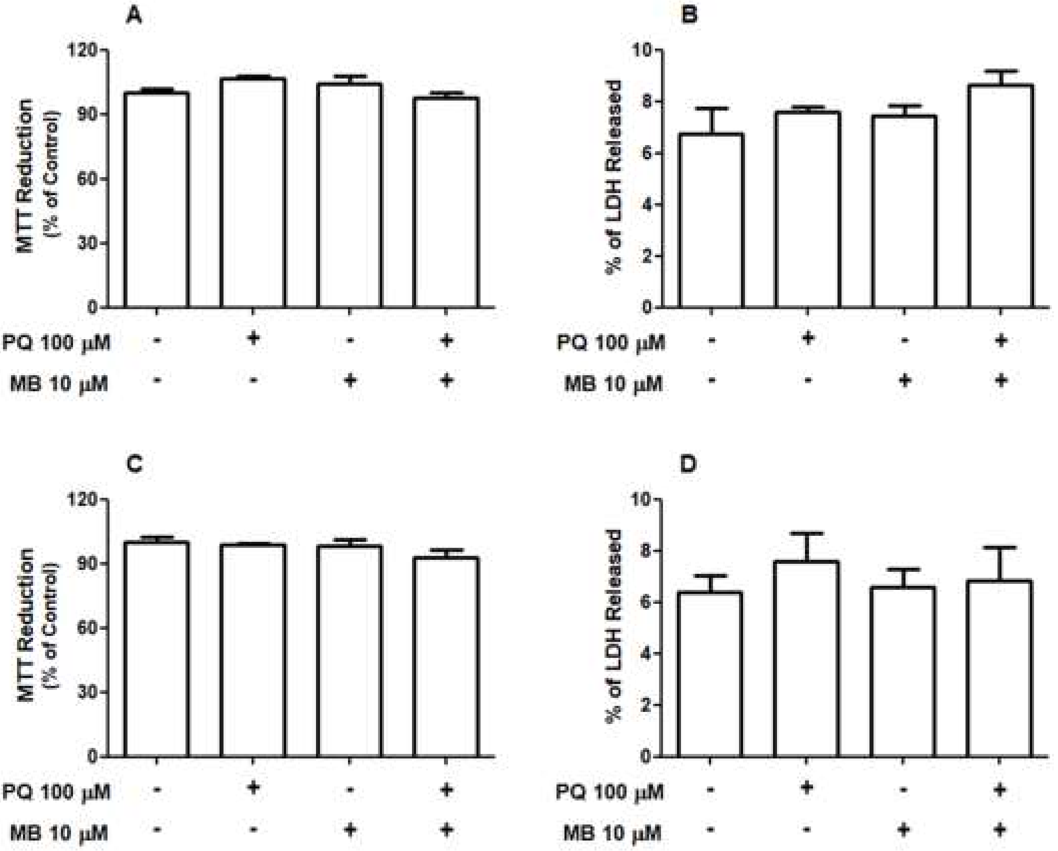

Legend. Cells were exposed to 100 µM PQ and 10 µM MB alone or in combination (PQ + MB) for 6 h (A and B) and 12 h (C and D). Cell viability was evaluated by the reduction of MTT (A and C) and by the LDH release assay (B and D). Results of MTT assays are expressed as the percentage of MTT reduction with respect to control values. Results of LDH release assays were expressed as percent of LDH released, where the 100% value represents control cells treated with 2% Triton X-100 for 15 min. Data are represented as mean ± S.E.M. (n= 5).

Figure S3. Morphological alterations in SH-SY5Y cells after PQ and/or MB exposure.

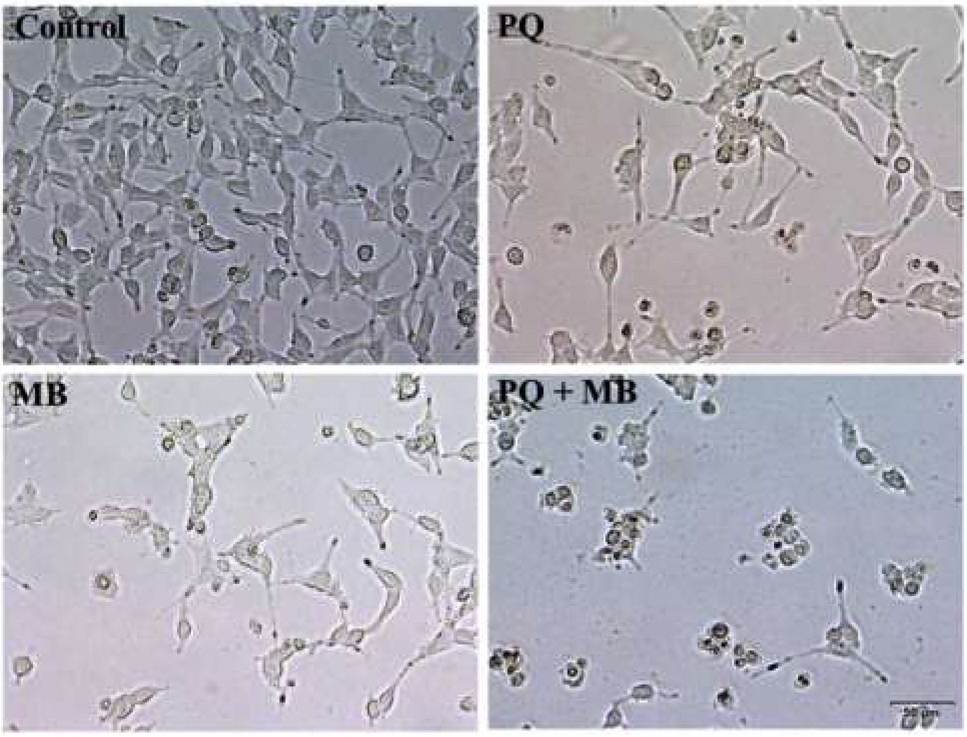

Legend. Cells were exposed to 100 μM PQ and 10 μM MB alone or in combination (PQ + MB) for 48 h. Representative images of the cells after pesticides exposures were taken in bright field in magnification of 200x (scale bar = 50 μm).

Figure S4. NAC treatment protects from PQ + MB-induced cytotoxicity in neuroblastoma SH-SY5Y cells.

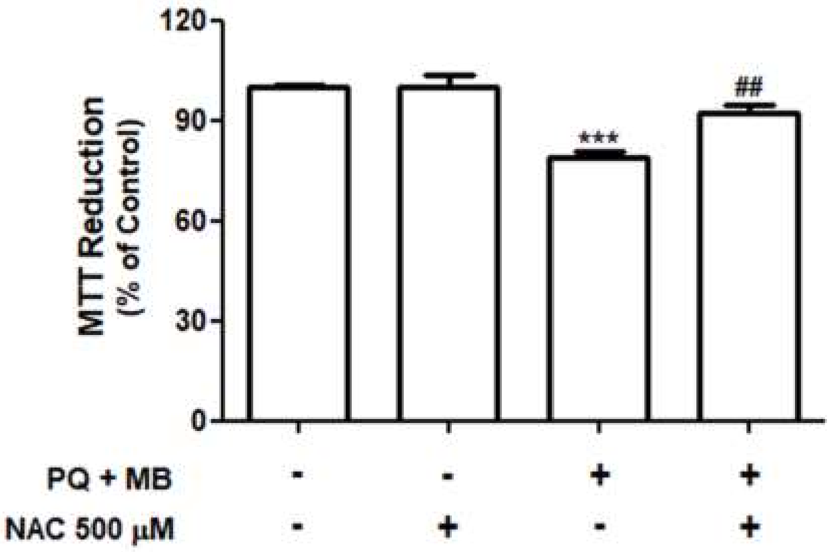

Legend. Cells were pretreated with NAC (500 µM) for 1 h and then exposed to 100 µM PQ and 10 µM MB in combination (PQ + MB) for 24 h. Cell viability was evaluated by the reduction of MTT and the results are expressed as the percentage of MTT reduction with respect to control values. Data are represented as mean ± S.E.M. (n= 4). *** $p<0.001$ indicates statistical difference from control and ## $p<0.01$ indicate statistical difference from PQ + MB by two-way ANOVA, followed by Tukey HSD post-hoc test.
